# Supplementary material for: Factors influencing the adoption and participation rate of nursing homes staff in a saliva testing screening programme for COVID-19
Source: PLoS One. 2022 Jun 30;17(6):e0270551. doi: 10.1371/journal.pone.0270551 (PMC9246163; doi:10.1371/journal.pone.0270551)
Supplement: Attachment — Submitted filename: PONE-D-22-00794_review.docx [file pone.0270551.s002.docx]

First of all, many thanks to the authors for their research in this relevant domain. I would like to give some comments/suggestions that could improve the manuscript.

1. **Introduction**

- Can you make the term 'prevention' concrete? Prevention of what is essential? Is it about prevention of transmission? Or is it prevention of hospital admissions or something else? This is of course all related, but it is not entirely clear to me in the first paragraph where the focus is in the manuscript.
- What about underlying diseases in elderly? This is also a reason why they were most heavily impacted by the SARS-CoV-2 pandemic. Unfamiliarity with the virus in the early stage of the pandemic will undoubtedly also have played a role…
- “Systematic screening of staff was highlighted as a priority as staff represent one of the main causes of the increased spread of COVID-19 in healthcare facilities.” 🡪 As this is stated in the introduction as being a priority, I would like to know where this info comes from. Please add a reference to this statement.
- “These questions have been poorly explored” 🡪 For what reasons?
- What exactly was the Walloon Region's proposal for systematic and gracious screening? Please elaborate further on this, as this is an important element of the study.
- I suggest putting the particular Walloon situation more in perspective by adding figures (e.g., amount of beds/1000 people; incidence and prevalence of infections; stats on test percentages among healthcare personnel; …). This makes it easier for international readers to frame the study and compare the situation described with theirs.
- Can you please clarify the link between the described psycho-sociological theories and the specific relevance of it in the study. How will these theories contribute to the outcome of the study?

2. **Materials and methods**

- I suggest structuring the Materials and methods section thoughtfully, e.g., 1) Study design and setting, 2) Study population, 3) Interventions and outcomes 4) Instruments, 5) Data analysis, and 6) Ethical considerations. A lot of information is currently being given, but somehow unstructured. Try to bring in a common thread here.
- Describing the context in terms of beds per 1000 people (🡪 can be found online) will give (international) readers a better insight in it. If you can find figures of the amount of healthcare personnel involved in the mentioned nursing homes, this will be also an added value to the manuscript.
- “The sample was taken and collected on the same day for all the workers of a same institution.” 🡪 This seems almost impossible to me. So please provide more details in your study about the study setting to put this in perspective.
- Please provide details about the test kits. Not only the manufacturer, but also the tested accuracy of the kit before the launch of it.
- Why was there a 3-week pilot phase of testing as the sample was taken and collected on the same day for all the workers. Or was this 3 week phase to give nursing homes the option to choose 1 day within a 3 week time frame? Please specify in the manuscript.
- Study population: How many employees in those 571 nursing homes (with differentiation between their backgrounds)? Please also list inclusion and exclusion criteria
- Data collection: Which questionnaire was used? Was it a new-developed one? Was it a validated questionnaire? What is meant by ‘standardized approach’? Did the employees have to agree individually with the transfer of their study results? Was there an informed consent? Please clarify all this or restructure the materials and methods section so it becomes clear for the readers.
- If important, add the questionnaire as supplementary material and therefore not in the body of the manuscript. Make it easy to read so it doesn't come across as course-like. Again, consider restructuring the materials and methods section of the paper
- Dependent variables (outcomes?): This should already be highlighted in the introduction or in a separate objectives section in the beginning of the materials and methods section. Why did you chose this outcome variables? Already refer to it somewhere in the introduction section.
- I could be wrong, but was a test schedule being used (as stated in the dependent variables section)?? I thought it was just a data collection at one point in time for every nursing home? Make this 100% clear throughout the manuscript.
- Ethical considerations: What happened with the consent of the individual employees (besides the consent on an institutional level)? Was it obtained? How was it obtained? This seems like an important element to me as personal data are transferred.

3. **Results**

- Participants: Is this (n = 571) all about independent nursing homes? Also be more specific in describing drop-out: How many due to unsuccessful contacts? When was a contact unsuccessful? When did you report a decision of non-participation? Was this just based on 1 phonecall? How is failure to contact defined? Can't find missing contact details online? How is ‘missing information defined’?

I have not found any data on the total number of employees who have enrolled the study? I think this is very important to mention.

4. **Discussion**

- “This figure demonstrates the extent to which saliva testing could be considered as a possible alternative to nasopharyngeal testing” 🡪 I do not agree with this conclusion, as the difference in accuracy between the two methods was not tested in the study. Maybe it is better to state it as an additional method.
- What exactly is done with the test results from the healthcare personnel?
