# Supplementary material for: Factors influencing the adoption and participation rate of nursing homes staff in a saliva testing screening programme for COVID-19
Source: PLoS One. 2022 Jun 30;17(6):e0270551. doi: 10.1371/journal.pone.0270551 (PMC9246163; doi:10.1371/journal.pone.0270551)
Supplement: Attachment — Submitted filename: Response to reviewers final version 16 5 22.docx [file pone.0270551.s003.docx]

***PONE-D-22-00794 Factors influencing the adoption and participation rate of nursing homes staff in a saliva testing screening programme for COVID-19***

**Response to Review**

We would like to thank the reviewers as well as the members of the Editorial Office for their revision of our manuscript. Below, you will find a point-by-point description of how each major comment was addressed in the manuscript.

**Journal Requirements:**

When submitting your revision, we need you to address these additional requirements.

1. Please ensure that your manuscript meets PLOS ONE's style requirements, including those for file naming. The PLOS ONE style templates can be found at

<https://journals.plos.org/plosone/s/file?id=wjVg/PLOSOne_formatting_sample_main_body.pdf> and

<https://journals.plos.org/plosone/s/file?id=ba62/PLOSOne_formatting_sample_title_authors_affiliations.pdf>

The manuscript has been adapted according to PLOS ONE’s style requirements

2. Please amend your current ethics statement to address the following concerns: Please explain how you recorded/documented participant consent, and if the ethics committees/IRBs approved this consent procedure.

As mentioned in our manuscript: “*The ethics committee of the University Hospital of Liège (Comité d’Ethique Hospitalo-Universitaire de Liège, Belgium) determined this study was exempt from review and did not oppose the undertaking of the study”.*

*🡪* Consequently, the ethics committee waived the recording of written participant consent. This information has been added in the manuscript.

As the participation was based on a voluntary basis and as the participation to the survey was made by phone call, we collected only oral consent from NH directors.

3. Thank you for stating the following financial disclosure:

“The funders had no role in study design, data collection and analysis, decision to publish, or preparation of the manuscript.”

At this time, please address the following queries:

a) Please clarify the sources of funding (financial or material support) for your study. List the grants or organizations that supported your study, including funding received from your institution.
b) State what role the funders took in the study. If the funders had no role in your study, please state: “The funders had no role in study design, data collection and analysis, decision to publish, or preparation of the manuscript.”
c) If any authors received a salary from any of your funders, please state which authors and which funders.
d) If you did not receive any funding for this study, please state: “The authors received no specific funding for this work.”

Please include your amended statements within your cover letter; we will change the online submission form on your behalf.

The correct statement regarding financial disclosure is d) “The authors received no specific funding for this work.” The cover letter has been amended accordingly.

4. PLOS requires an ORCID iD for the corresponding author in Editorial Manager on papers submitted after December 6th, 2016. Please ensure that you have an ORCID iD and that it is validated in Editorial Manager. To do this, go to ‘Update my Information’ (in the upper left-hand corner of the main menu), and click on the Fetch/Validate link next to the ORCID field. This will take you to the ORCID site and allow you to create a new iD or authenticate a pre-existing iD in Editorial Manager. Please see the following video for instructions on linking an ORCID iD to your Editorial Manager account: <https://www.youtube.com/watch?v=_xcclfuvtxQ>

ORCID Benoit Pétré: 0000-0002-5344-4401

My profile has been updated.

5. Please include captions for your Supporting Information files at the end of your manuscript, and update any in-text citations to match accordingly. Please see our Supporting Information guidelines for more information: <http://journals.plos.org/plosone/s/supporting-information>.

Supporting information

S1 Table. Underlying data set for the study of factors influencing the adoption and participation rate of nursing homes staff in a saliva testing screening programme for COVID-19

(XLSX)

**Reviewer #1:**

Many thanks to the authors for their research in this relevant domain. My specific comments/suggestions can be found in the attached file. In general, the framing in the introduction still needs to be refined, as well as the structuring of the reporting in the Materials and Methods section.

First of all, many thanks to the authors for their research in this relevant domain. I would like to give some comments/suggestions that could improve the manuscript.

1. **Introduction**

- Can you make the term 'prevention' concrete? Prevention of what is essential? Is it about prevention of transmission? Or is it prevention of hospital admissions or something else? This is of course all related, but it is not entirely clear to me in the first paragraph where the focus is in the manuscript.

Indeed, prevention is both individual and collective in such pandemic context. We changed the first paragraph as following: “Prevention is essential both for individuals and community in order to prevent the spread of the virus, avoid saturation of health care services and also to combat the psycho-medico-social consequences of the crisis. Therefore, in addition to individual prevention measures, early detection, triage and effective isolation of potentially infected and infectious patients are essential to prevent unnecessary community exposure (1) and to break the chains of transmission”

- What about underlying diseases in elderly? This is also a reason why they were most heavily impacted by the SARS-CoV-2 pandemic. Unfamiliarity with the virus in the early stage of the pandemic will undoubtedly also have played a role…

We know that nursing home residents are multimorbid, immunocompromised and frail - and therefore most vulnerable to complications from COVID-19. These characteristics have been added in the introduction section.

- “Systematic screening of staff was highlighted as a priority as staff represent one of the main causes of the increased spread of COVID-19 in healthcare facilities.” 🡪 As this is stated in the introduction as being a priority, I would like to know where this info comes from. Please add a reference to this statement.

This is reported in:

- European Centre for Disease Prevention and Control. Surveillance of COVID-19 in long-term care facilities in the EU/EEA, 19 May 2020. Stockholm: ECDC; 2020.

This is especially true when visits of residents are suspended or strictly limited. See:

- Wilmes et al. SARS-CoV-2 transmission risk from asymptomatic carriers: results from a mass screening promamme in Luxembourg. Lancet Reg. Health Eur. 4, 100056 (2021)
- Vandael E, Latour K, Islamaj E, Panis L, Callies M, Haarhuis F et al. COVID-19 cases, hospitalizations and deaths in Belgian nursing homes: results of a surveillance conducted between April and December 2020. Arch Public Health. 2022;80(1).

References have been also added in the manuscript.

- “These questions have been poorly explored” 🡪 For what reasons?

The panic that emerged from the pandemic reactivated paternalistic and biomedical approach of governance that put health promotion on the back burner over the crisis (mainly at the beginning). Cambon L, Bergeron H, Castel P, Ridde V, Alla F. When the worldwide response to the COVID-19 pandemic is done without health promotion. Glob Health Promot. 2021;175797592110151.

- What exactly was the Walloon Region's proposal for systematic and gracious screening? Please elaborate further on this, as this is an important element of the study.

The Walloon Government is responsible for the health of elderly, particularly the management of Nursing homes. In view of the disastrous situation in Belgium with regard to COVID cases, the Walloon Government wished to help reduce the burden on the MR/MRS and financially supported: the provision of saliva sampling kits; the analysis of samples by PCR; the delivery of results; and the logistical operations necessary for the implementation of the system. However, this evaluation study was not funded by the Walloon Government and was conducted completely independently by the authors of the paper. The results were communicated to the government in order to adjust and improve the strategy for implementing testing.

We have injected some additional information in the context section.

- I suggest putting the particular Walloon situation more in perspective by adding figures (e.g., amount of beds/1000 people; incidence and prevalence of infections; stats on test percentages among healthcare personnel; …). This makes it easier for international readers to frame the study and compare the situation described with theirs.

Belgium has a high proportion of aging population with approximately 19% of the population older than 65 years. 5.7% of the latter group is living in NHs. Belgium has one of the highest number of NH beds per 1000 population in Europe (50 NH beds per 1000 population over 60) and tops the chart with the number of people of 85 years and older living in an institution. Belgium was also ranked among the countries with the highest COVID-19 mortality in the world during its first COVID-19 wave.

We had some explanations in introduction section and also refer to the following ressources for more details on COVID 19 EPIDEMIC in NH in Belgium

- Vandael E, Latour K, Islamaj E, Panis L, Callies M, Haarhuis F et al. COVID-19 cases, hospitalizations and deaths in Belgian nursing homes: results of a surveillance conducted between April and December 2020. Arch Public Health. 2022;80(1).
- Gillain S, Belche JL, Moreau JF. COVID-19 epidemic in the nursing homes in Belgium. Jour Nursing Home Res. 2020;6:40-42
- Can you please clarify the link between the described psycho-sociological theories and the specific relevance of it in the study. How will these theories contribute to the outcome of the study?

To the best of our knowledge’s, there is no theories that explain the (non) adoption of health preventive behaviours against covid spreading. Based on generic psycho-sociological theories, we aimed to identify which factors could explain the (non) participation in preventative health practices in order to adjust health prevention strategies.

2. **Materials and methods**

- I suggest structuring the Materials and methods section thoughtfully, e.g., 1) Study design and setting, 2) Study population, 3) Interventions and outcomes 4) Instruments, 5) Data analysis, and 6) Ethical considerations. A lot of information is currently being given, but somehow unstructured. Try to bring in a common thread here.

We revised partially the structure of the section “Materials and methods”

- Describing the context in terms of beds per 1000 people (🡪 can be found online) will give (international) readers a better insight in it. If you can find figures of the amount of healthcare personnel involved in the mentioned nursing homes, this will be also an added value to the manuscript.

The amount of health care personnel is estimated over 35.000 for studied nursing home. Information added in the manuscript.

- “The sample was taken and collected on the same day for all the workers of a same institution.” 🡪 This seems almost impossible to me. So please provide more details in your study about the study setting to put this in perspective.

This is one of the limit of the study due to the general organization of saliva testing for the Walloon nursing homes during the pilot study. A unique time slot was dedicated for each nursing home for the collection of salivary samples. It is obviously one of the main reason explaining limited observed participation of staff. It is more discussed in limits section

- Please provide details about the test kits. Not only the manufacturer, but also the tested accuracy of the kit before the launch of it.

See for a more detailed description of the kit:

-Donneau A, Guillaume M, Bours V, Dandoy M, Darcis G, Desmecht D et al. University population-based prospective cohort study of SARS-CoV-2 infection and immunity (SARSSURV-ULiège): a study protocol. BMJ Open. 2022;12(1):e055721

-Saegerman C, Diep A, Renault V, Donneau A, Stamatakis L, Coppieters W et al. A 2-month field cohort study of SARS-CoV-2 in saliva of BNT162b2 vaccinated nursing home workers. Communications Medicine. 2022;2(1).

The references have been added.

- Why was there a 3-week pilot phase of testing as the sample was taken and collected on the same day for all the workers. Or was this 3 week phase to give nursing homes the option to choose 1 day within a 3 week time frame? Please specify in the manuscript.

Because recommendation claim for a weekly testing for staffs. The idea was that each member of the staff has to test himself each week on the dedicated day during the three weeks of the pilot phase.

- Study population: How many employees in those 571 nursing homes (with differentiation between their backgrounds)? Please also list inclusion and exclusion criteria

The screening involved all employed staff, self-employed staff working in the institution, trainees and volunteers (= staff members) (from nurses to technicians). Staff members were invited to be tested weekly on a voluntary basis.

- Data collection: Which questionnaire was used? Was it a new-developed one? Was it a validated questionnaire? What is meant by ‘standardized approach’?

This is a home made questionnaire inspired on existing one’s. The point is now mentioned in the methods section.

The “Standardized approach” was for the interviewers in order to collect date according the same approach.

- If important, add the questionnaire as supplementary material and therefore not in the body of the manuscript. Make it easy to read so it doesn't come across as course-like. Again, consider restructuring the materials and methods section of the paper

We already add the row data as supplementary material and a clear description of how the data have been collected is already present in the method section.

Dependent variables (outcomes?): This should already be highlighted in the introduction or in a separate objectives section in the beginning of the materials and methods section. Why did you chose this outcome variables? Already refer to it somewhere in the introduction section.

The selection of this outcome is mainly motivated by available data. As already mentioned, this evaluation study was independent from the screening program put in place. We had to base our selection on what was collected through the screening program.

- I could be wrong, but was a test schedule being used (as stated in the dependent variables section)?? I thought it was just a data collection at one point in time for every nursing home? Make this 100% clear throughout the manuscript.

You are right: the data collection was at one point in time for EACH nursing home over the week. A specific time slot was dedicated for each NH. But the different nursing homes had different moments of saliva samples collection. It has been clarified in BOX 1.

- Did the employees have to agree individually with the transfer of their study results? Was there an informed consent? Please clarify all this or restructure the materials and methods section so it becomes clear for the readers.Ethical considerations: What happened with the consent of the individual employees (besides the consent on an institutional level)? Was it obtained? How was it obtained? This seems like an important element to me as personal data are transferred.

The testing device has been designed to deliver the result of the samples in a completely anonymous way. The result of an analysis is delivered on presentation of a barcode number of the corresponding sample, without being associated with an identity. Consequently, only the person who has taken a self-sample of saliva and who has the corresponding barcode number can make the link between him or herself and the result delivered on the interface (website). Only statistical, aggregated information was communicated to the organisation that requested the tests. There is no personal data that are transferred.

Neither the Walloon Region, nor the Nursing Home authorities, nor the University of Liège are able to link a sample to the person concerned; nor are they able to determine which persons have or have not taken part in regular tests. It was therefore not possible to request nominative consent.

Nevertheless, when consulting the results via the computer interface, persons who had submitted a sample and wished to know the result of the analysis were informed about the uses of their salivary testing to guide the following objectives:

1. To enable all persons who have submitted a saliva sample to find out the result of the test;

2. To enable the testing laboratory to inform the third party organisation, in a statistical and completely anonymous manner, of the degree of infection of its members;

3. To carry out statistics and analyses to evaluate the effectiveness of the system;

4. Carry out anonymous epidemiological research

Some information has been added in ethical aspects of the study.

3. **Results**

- Participants: Is this (n = 571) all about independent nursing homes? Also be more specific in describing drop-out: How many due to unsuccessful contacts? When was a contact unsuccessful? When did you report a decision of non-participation? Was this just based on 1 phonecall? How is failure to contact defined? Can't find missing contact details online? How is ‘missing information defined’?

Details are shown in 2.6. “Organisation of data collection”. However, figure 1 has been completed to clarify dropout aspects.

I have not found any data on the total number of employees who have enrolled the study? I think this is very important to mention.

The whole staff from studied nursing homes have been invited to enroll the study. Number of people who finally enrolled the study is given by the participation rate (see level of staff engagement).

4. **Discussion**

- “This figure demonstrates the extent to which saliva testing could be considered as a possible alternative to nasopharyngeal testing” 🡪 I do not agree with this conclusion, as the difference in accuracy between the two methods was not tested in the study. Maybe it is better to state it as an additional method.

The suggestion has been integrated in the text

- What exactly is done with the test results from the healthcare personnel?

It is explain in the step 5 of the procedure of testing: “The results could be obtained in two ways: the person who took the sample could obtain his/her individual result by entering the recorded barcode number of their sample (anonymity was respected, since only the participant knew their barcode), or the nursing homes direction could consult a statistical report of anonymized and aggregated results for their establishment”

**Reviewer #2**:

- This is a report of nation (region) - wide survey of managers and staff of nursing homes in Wallonia (french-speaking part of) Belgium. The study explores the willingness to participate of nursing home managers in a preventive testing programme, using systematic and repeated (during a 3 months period) of saliva-self-tests of staff members during the COVID pandemic. In addition, the real implementation of the programme is explored, using he data on actually performed self-tests.The study is well powered, almost a full population study, well designed and conducted, and providing usefull information for health policy makers, responsible for pandemic response in this particular sector. The discussion is to the point and well indicating the limitations. The conclusions are within the realm of the findings.
  The authors may add more information about the evidence on sensitiviry and speciicity of the selected saliva-seff-test.

The quality of saliva self test has been described previously. We have added a description and reference in the manuscript.
